# Excellent Outcome of Protocol-Based Treatment of Motivated Type 2 Diabetes Mellitus (T2DM) Patients: A Real World Follow-Up for 11 Years

**DOI:** 10.7759/cureus.11558

**Published:** 2020-11-18

**Authors:** Sudip Chatterjee, Anirban Majumder

**Affiliations:** 1 Endocrinology, Vivekananda Institute of Medical Sciences, Kolkata, IND; 2 Endocrinology, Kali Prasad Chowdhury Medical College & Hospital, Kolkata, IND

**Keywords:** t2dm, ada guidelines, long term follow up, outcome, real world

## Abstract

Introduction and aim

To evaluate the real-world clinical outcome of guideline-based treatment among adherent and committed type 2 diabetes mellitus (T2DM) patients.

Methods

The study reports the outcomes of an 11-year clinic-based standard care regime, based on the American Diabetes Association (ADA) guidelines and implemented in the authors’ practices. Records of 145 T2DM patients, who were regularly followed up, were reviewed. Descriptive and inferential statistical analysis was carried out with the Statistical Analysis System (SAS) (SAS Institute Inc., Cary, NC, USA) and with Statistical Package for Social Sciences (SPSS) (IBM Corp., Armonk, NY, USA), with Microsoft Word and Excel to generate graphs and tables.

Results

Apart from a significant increase of body weight (but not of body mass index, BMI) and a significant decrease of diastolic blood pressure (DBP), there were insignificant changes in all major biochemical parameters, fasting plasma glucose (FPG), postprandial plasma glucose (PPG), glycated hemoglobin (HbA1c), low-density lipoprotein cholesterol (LDL-C), creatinine, estimated glomerular filtration rate (eGFR) and urine albumin creatinine ratio (ACR), over the 11 years of follow-up.

Conclusion

ADA guideline-based management effectively maintained treatment goals among treatment adherent and committed T2DM patients over 11 years. Glycemic parameters (FPG, PPG, and HbA1c) and renal parameters (serum creatinine, eGFR, and ACR levels) remained stable. Our outcomes data were better than those recorded in the landmark United Kingdom Prospective Diabetes Study (UKPDS) and Action in Diabetes and Vascular Disease: Preterax and Diamicron Modified Release Controlled Evaluation (ADVANCE).

## Introduction

The number of people with diabetes is growing globally [1]. The landmark United Kingdom Prospective Diabetes Study (UKPDS) had shown that improvement in blood glucose control reduced the risk of diabetes-related complications and multiple interventions need to be initiated early to prevent the development of complications in persons with diabetes [2]. Post-UKPDS, the management of type 2 diabetes mellitus (T2DM) conventionally followed a stepwise approach. The American Diabetes Association's (ADA) clinical practice guidelines provide components of diabetes care and treatment goals and are published yearly [3,4]. Management commences with lifestyle intervention, followed by the addition of a single oral anti-diabetic drug, usually metformin, then a combination of oral drugs and eventually insulin. Many new oral anti-diabetic agents and designer insulin have been developed over the last 15 years. Despite the availability of evidence-based regularly updated guidelines [3,4], many patients cannot maintain the recommended levels of glycaemic control [5]. There is however a small subset of patients who are regular in their follow-up and diligent in their adherence to the treatment guidelines. We have retrospectively evaluated the outcome of the guideline-based treatment protocol in our adherent patients over 11-year period of follow-up.

## Materials and methods

Study population

We reviewed the records of 1031 patients with T2DM who attended our practices between January 2007 and December 2017.

Inclusion criteria:

1. Age above 20 years on January 1, 2007 2. Diagnosed as T2DM 3. Enrolled in the practice by Jan 31, 2007 4. Continuing in the practice up to Dec 31, 2017 5. Attended follow-up clinic visit at least twice each year between 2007 and 2017.

We were unable to ascertain the number of deaths that occurred during this period. There were 145 patients who met the criteria.

Management protocol

Our treatment protocols followed the most recent ADA guidelines and hence changed over the years. Drug use followed the broad guidelines laid down by ADA which gives considerable leeway to the clinician to choose between different classes of drugs. On scrutiny of individual patient data, we did not find any consistent pattern favoring or disfavoring any drug class.

Data collection

Data were collected from electronic records regarding demographic information (age, sex, duration of diabetes, smoking history), body weight, body mass index (BMI), systolic blood pressure (SBP), diastolic blood pressure (DBP), fasting plasma glucose (FPG), postprandial plasma glucose (PPG), glycated hemoglobin (HbA1c) levels, low-density lipoprotein cholesterol (LDL-C), serum creatinine, estimated glomerular filtration rate (eGFR), and urine albumin creatinine ratio (ACR). Data pertaining to eye and foot examination and history of cerebrovascular disease (CVA), cardiovascular disease and cardiac interventions (CVD), and peripheral vascular disease (PVD) were also recorded. CVD included coronary artery disease (CAD), myocardial infarction (MI), percutaneous coronary intervention (PCI), and coronary artery bypass grafting (CABG). Data were collected from electronic records of 2007 (baseline) and of 2017 (end of observation). The study protocol was presented to the Ethics Committee of one of the authors’ institutions, who opined that given the retrospective chart review proposed, no informed consent was needed.

Outcome measures

The primary outcome of the study was to determine differences in HbA1c between 2007 and 2017. Secondary outcomes included changes in physical parameters (weight, BMI, SBP, and DBP), changes in laboratory values (FPG & 2-hour PPG, HbA1c, LDL-C, serum creatinine, and eGFR), and change in the incidence of diabetes complications (number of individuals with eGFR <60, number of individuals with urine ACR >300, neuropathy, retinopathy, CVA, CVD, and PVD). Neuropathy was defined as impaired or absent vibration perception (as measured routinely by biothesiometer) or monofilament test (as measured routinely by Semmes Weinstein 10 gm monofilament) over the malleoli and tip of the big toe. Retinopathy was defined as new macular edema, proliferative or non-proliferative retinopathy, blindness, use of laser photocoagulation, or intravitreal injection.

Statistical analysis

Descriptive and inferential statistical analysis was carried out with Statistical Analysis System (SAS, version 9.2 for Windows, SAS Institute Inc., Cary, NC, USA) and with Statistical Package for Social Sciences (SPSS, Complex Samples, version 21.0 for Windows, IBM Corp., Armonk, NY, USA), along with Microsoft Word and Excel to generate graphs and tables. The study variables which were parametric were expressed as mean and standard deviation, while those following non-parametric distribution were expressed as a median and inter-quartile range. The data on categorical variables were presented as actual numbers and percentages. We used a paired t-test for comparing the parametric data and Wilcoxon signed-rank test for non-parametric data. To compare complications of diabetes we used McNemar Exact probability and McNemar Chi-squared tests. The statistical significance was assessed at a level of 5%.

## Results

Only 14% (145 out of 1031) patients from our records in January 2007 completed an 11-year follow-up and satisfied the inclusion criteria. Their mean age was 55.47 years and the mean duration of diabetes was 7.14 years in 2007. Of these 145 patients, 80 (55%) were men and 65 (45%) were women. None were smokers (Table 1).

**Table 1:** Baseline characteristics as on 2007 (n = 145) BMI: Body mass index; SBP: Systolic blood pressure; DBP: Diastolic blood pressure; FPG: Fasting plasma glucose; PPG: Postprandial plasma glucose; HbA1c: Glycated hemoglobin; LDL-C: Low-density lipoprotein cholesterol; eGFR: estimated glomerular filtration rate.

Parameter	Value
Age (in years), Mean ± SD	55.47 ± 09.78
Male, n (%)	80.00 (55.17)
Duration of Diabetes (in years), Mean ± SD	7.14 ± 6.97
Weight (kg), Mean ± SD	65.90 ± 11.72
BMI (kg/m^2^), Mean ± SD	25.65 ± 4.48
SBP (mmHg), Mean ± SD	135.13 ± 17.46
DBP (mmHg), Mean ± SD	80.96 ± 10.18
FPG (mmol/L), Mean ± SD	8.27 ± 2.92
PPG (mmol/L), Mean ± SD	11.86 ± 04.48
HbA1c (%), Mean ± SD	7.64 ± 1.26 (60.00 ± 13.80 mmol/mol)
LDL-C (mmol/L), Mean ± SD	2.76 ± 00.86
Creatinine (μmol/L), Mean ± SD	87.54 ± 25.64
eGFR (mL/min/1.73 m²), Mean ± SD	86.25 ± 23.24

Over 11 years, statistically significant weight gain occurred from 65.90 ± 11.72 kg to 67.46 ± 11.62 kg but without any statistically significant change in BMI. The decrease in SBP from 135.13 ± 17.46 mmHg to 133.27 ± 15.31 mmHg was not statistically significant but a drop in DBP from 80.96 ± 10.18 to 76.23 ± 9.81 was statistically significant (Table 2).

**Table 2:** Changes in the anthropometric parameters from 2007 to 2017 (n = 145) SBP: Systolic blood pressure; DBP: Diastolic blood pressure.

Physical Parameters	2007 (mean ± SD)	2017 (mean ± SD)	P (Paired t-test)
Weight (kg)	65.90 ± 11.72	67.46 ± 11.62	0.001
BMI	25.65 ± 4.48	26.04 ± 4.35	0.102 (NS)
SBP (mm Hg)	135.13 ± 17.46	133.27 ± 15.31	0.214 (NS)
DBP (mm Hg)	80.96 ± 10.18	76.23 ± 09.81	<0.001

All patients showed improvements on the FPG, PPG, HbA1c, and LDL-C measures over 11-year follow-up but not significantly. There was a mild deterioration of the renal parameter as the mean creatinine value went up to 1.07 mg/dl from 0.99 mg/dl but also not significant (Table 3).

**Table 3:** Changes in the biochemical parameters from 2007 to 2017 (n = 145) FPG: Fasting plasma glucose; PPG: Postprandial plasma glucose; HbA1c: Glycated hemoglobin; LDL-C: Low-density lipoprotein cholesterol; eGFR: estimated glomerular filtration rate.

Laboratory Parameters	2007 (mean ± SD)	2017 (mean ± SD)	P (Paired t-test)
FPG	8.27 ± 2.92	7.76 ± 2.57	0.867 (NS)
PPG	11.86 ± 4.48	10.30 ± 3.42	0.991 (NS)
HbA1c	7.64 ± 1.26 (60.00 ± 13.80)	7.53 ± 1.29 (59.00 ± 14.00)	0.154 (NS)
LDL-C	2.76 ± 00.86	2.03 ± 00.78	0.086 (NS)
Creatinine	87.54 ± 25.64	94.61 ± 40.67	0.086 (NS)
eGFR	86.25 ± 23.24	78.15 ± 20.36	0.551 (NS)

An insignificant rise in the ACR was observed over 11 years (Table 4).

**Table 4:** Changes in the spot urine albumin to creatinine ratio (ACR) between 2007 and 2017 (n = 80)

ACR (mcg/L of albumin to mg/L of creatinine)	Median	Interquartile range (IQR)	p-value computed by Wilcoxon Signed Rank test
In 2007	18.50	7.30-26.70	0.682 (NS)
In 2017	25.60	10.80-30.87	

Parameters like FPG and 2-hour PPG, HbA1c, LDL-C, eGFR, blood pressure, and BMI were held at target, there was a significant rise in macro and microvascular complications in this cohort over the 11 years’ of follow-up (Table 5).

**Table 5:** Micro and macrovascular complications of the patients CVA: Cerebrovascular disease; CVD: Cardiovascular disease & cardiac interventions; PVD: Peripheral vascular disease; eGFR: estimated glomerular filtration rate; ACR: Albumin to creatinine ratio.

Parameters of diabetes complications	Year 2007	Year 2017	p-value (from McNemar’s test)
eGFR < 60, n (%)	23 (15.9%)	39 (26.9%)	0.006
Urine ACR >300, n (%)	5 (3.44%)	9 (6.20%)	0.388
Neuropathy, n (%)	6 (4.13%)	19 (13.1%)	0.001
Retinopathy, n (%)	00.00	8 (5.51%)	0.008
CVA, n (%)	00.00	1 (0.68%)	1.000
CVD, n (%)	2 (1.37%)	10 (6.89%)	0.008
PVD, n (%)	00.00	7 (4.82%)	0.016

## Discussion

In this study, the effect of 11 years of specialist-based, targeted, multi-intervention care with lifestyle and intensification of pharmacological treatment was evaluated to determine its impact on glycemic control, physical parameters, biochemical parameters, and diabetes complications. It was not possible to collect data on patients who chose not to come for follow-up. A 3-point major adverse cardiovascular event (MACE) could not be determined as we could not reliably count the number of deaths that may have occurred. It was possible that only the well-controlled patients came for regular follow-up. The patient recollection of hypoglycaemic episodes is known to be poor and hence was not recorded [6].

Both practices followed ADA guidelines as amended from time to time [3,4]. This helped to provide a consistent standard of care. It was not possible to compare the ADA model with other diabetes care models in the present study. HbA1c, the primary outcome measure, did not change over time but remained markedly lower than the Indian average of 8.91% [7].

We found a significant increase in body weight (but not in BMI) and a significant decrease in DBP (but not in SBP). There was no significant change in blood biochemistry (FPG, PPG, HbA1c, LDL-C, and serum creatinine). So the glycemic parameters (FPG, PPG, and HbA1c) and renal parameters (serum creatinine, eGFR, and ACR levels) remained stable over 11 years. However, we observed an increase in the number of individuals with eGFR <60, the number of individuals with urine ACR >300, neuropathy, retinopathy, CVA, CVD, and PVD.

A small sample size precluded further analysis. Our cohort of patients who came for regular follow-up (at least twice each year) and adhered to ADA guidelines constituted a minority (14%) amongst the T2DM patients treated by us. However, outcomes in this cohort were better than those reported in the literature. UKPDS showed that diabetes progressed irrespective of efforts to control blood glucose and the proportion of patients achieving control declined over time, requiring the use of additional medications [2]. Although the UKPDS used diabetes control measures available in the 1980s, the study enrolled only newly diagnosed patients. The UKPDS did not achieve stable glucose levels. The HbA1c level rose from a median of 6.6% (48.6 mmol/mol) to 8.1% (65 mmol/mol) in the intensive treatment group with a 0.9% separation in median HbA1c from the standard treatment group throughout the 10-year study. There was no difference in the rise of the HbA1c level with respect to the type of treatment used. Microvascular complications also rose progressively but were 12% lower in the intensive group compared to the standard group without any glycemic threshold for any of the complications. In our group, HbA1c and major biochemical values remained unchanged over 11 years despite the fact that our patients had a mean duration of diabetes of 7.14 years at study entry. However, we noted a rise of microangiopathy (numerical increase in the number of individuals with eGFR <60, number of individuals with urine ACR >300, neuropathy, and retinopathy) in our patients, despite consistent adherence to protocol. This seems to represent a residual risk in our patients, analogous to the concept of residual risk in preventive cardiology [8].

There was no consistent pattern to the second drug use after metformin. ADA guidelines between 2007 and 2017 stipulated that any drug or insulin could be used after metformin. Between 2007 and 2017 numerous novel but costly antidiabetic medications and insulins were launched in India. ADA’s advice regarding preferential use of Sodium-glucose co-transporter2 (SGLT2) inhibitors or Glucagon-like peptide-1 receptor agonists (GLP1RA) in patients with known or suspected atherosclerotic cardiovascular disease (ASCVD) was first given in 2018 and was not applicable to this cohort. Four dipeptidyl peptidase-4 (DPP4) inhibitors (sitagliptin, vildagliptin, saxagliptin, and linagliptin), were launched between 2008 and 2012. GLP-1RAs (liraglutide, lixisenatide, and dulaglutide) became available between 2007 and 2014. SGLT2 inhibitors were launched in 2015 in India [9]. Our patients received all the available drugs including insulin, but none were on GLP-1 RA’s. Long-acting basal insulins (glargine, detemir, and degludec) became available in 2003, 2006, and 2013, respectively. The first insulin use in our cohort was invariably either glargine or degludec. Both these insulins are known to significantly improve glycemic control and reduce the risk of hypoglycemia, without increasing body weight [10].

Perhaps due to the availability of many new, safe, and effective agents, glycemic control in our cohort did not change (7.64% vs 7.53% or 60 vs 59 mmol/mol) over 11 years. Further, the eGFR (86.25 ± 23.24 in 2007 vs 78.15 ± 20.36 in 2017), urine ACR (18.5 in 2007 vs 25.6 in 2017) and serum creatinine level (87.54 umol/L in 2007 vs 94.61 umol/L in 2017), remained stable. The small increase in the number of patients with eGFR <60 and the number of patients with ACR >300 was not significant. The other renal outcomes (creatinine and urine ACR) were better than what is reported in the literature. The progressive decline in renal function over 10 years was well documented in UKPDS, showing progression to microalbuminuria at 2.0% per year from the diagnosis of diabetes, from microalbuminuria to macroalbuminuria at 2.8% per year, and from macroalbuminuria to elevated plasma creatinine at 2.3% per year [11].

Action in diabetes and vascular disease

Preterax and Diamicron Modified Release Controlled Evaluation (ADVANCE) trial recruited patients with a mean duration of 7.9 years and followed them for a median of five years [12]. The intensive treatment group maintained HbA1c of 6.5% (47.5 mmol/mol). In this group, 9.4% had microvascular complications. There was worsening neuropathy in 42.2%, worsening nephropathy in 4.1% (less than in the control group), and worsening eye disease in 6% [12]. In view of the progressive nature of T2DM, as evidenced from UKPDS and ADVANCE, our real-world data showed a better outcome for microangiopathy (Table 5). In the absence of a control arm, we were unable to quantify the benefits achieved in other micro- and macrovascular complications of diabetes.

Our study had several strengths and limitations. The strength of the study lies in its presentation of real-world data on glycemic control, biochemical parameters, and microvascular outcomes in protocol adherent patients. In our fee for the treatment model, patients determine the frequency of follow-up and are free to change their physician at any time. Most of our patients either did not come for follow-up or were irregular, with less than two clinic visits a year, and were ineligible for this study. Approximately 14% (145 out of 1031) of the patients in our records in January 2007 remained active by the end of 2017. We do not know why most of our patients became inactive. It is possible that only well-controlled patients chose to remain in our practices.

The study was not population-based and selection bias is self-evident. The study exclusively included two private practices in one city of India and hence, our data cannot be generalized to the larger diabetes community of India. The sample size was small and no control group was enrolled. We had no data on the type of diabetes control the patients had before coming under our care. We could not calculate the standard 3-point MACE in our patients as we had no information on patient deaths. SGLT-2 inhibitor class of drugs with proven renal and cardiovascular benefits were available for a very short period towards the end of the study only and GLP1RAs were not used. Data on urinary ACR remained incomplete in this retrospective study. However, our study encapsulated the benefits attainable in a real-world setting with motivated patients receiving guideline-based care. Long follow-up of patients remains a challenge in our setting, but can be achieved. In spite of excellent adherence, our patients were not totally free of micro- and macrovascular complications. This suggests that a degree of residual risk for complications persists in T2DM patients.

## Conclusions

This study provides strong real-world evidence of the effectiveness of structured ADA-based management in people with T2DM in India. It supplements existing randomized control trials (RCTs). Patients who were adherent and committed could maintain good control over diabetes. This was undoubtedly aided by advances in diabetes management over the years. Over an 11-year period, complications increased even in this motivated group. This possibly shows that with our existing tools, we cannot completely eliminate the complication burden of diabetes.
